# Supplementary material for: Rapid discovery of self-assembling peptides with one-bead one-compound peptide library
Source: Nat Commun. 2021 Jul 23;12:4494. doi: 10.1038/s41467-021-24597-5 (PMC8302598; doi:10.1038/s41467-021-24597-5)
Supplement: Supplementary file 1 — Supplementary Information [file 41467_2021_24597_MOESM1_ESM.pdf]

Supplementary Information

Rapid Discovery of Self-Assembling Peptides with One-Bead One-Compound Peptide Library

Pei-Pei Yang[1,#], Yi-Jing Li[1,2,#], Yan Cao[3,#], Lu Zhang[4], Jia-Qi Wang[1], Zi-Wei Lai[3], Kuo Zhang[1,2], Diedra Shorty[4], Wenwu Xiao[4], Hui Cao[2], Lei Wang[1,]*, Hao Wang[1,5,]*, Ruiwu Liu[4,]*, Kit S. Lam[4,6,]*

[1]CAS Center for Excellence in Nanoscience, CAS Key Laboratory for Biomedical Effects of Nanomaterials and Nanosafety, National Center for Nanoscience and Technology (NCNST) No. 11 Beiyitiao, Zhongguancun, Beijing, 100190, China.
[2]Department of Materials Physics and Chemistry, School of Materials Science and Engineering, University of Science and Technology Beijing, Beijing, 100083, P. R. China.
[3]Institute for Advanced Study, Shenzhen University, Guangdong 518060, China.
[4]Department of Biochemistry and Molecular Medicine, UC Davis NCI-designated Comprehensive Cancer Center, University of California Davis, Sacramento, California 95817, United States.
[5]Center of Materials Science and Optoelectronics Engineering, University of Chinese Academy of Sciences, Beijing 100049, P. R. China.
[6]Division of Hematology and Oncology, Department of Internal Medicine, School of Medicine, University of California Davis, Sacramento, California 95817, United States.

E-mail: kslam@ucdavis.edu; rwliu@ucdavis.edu; wanglei@nanoctr.cn; wanghao@nanoctr.cn.

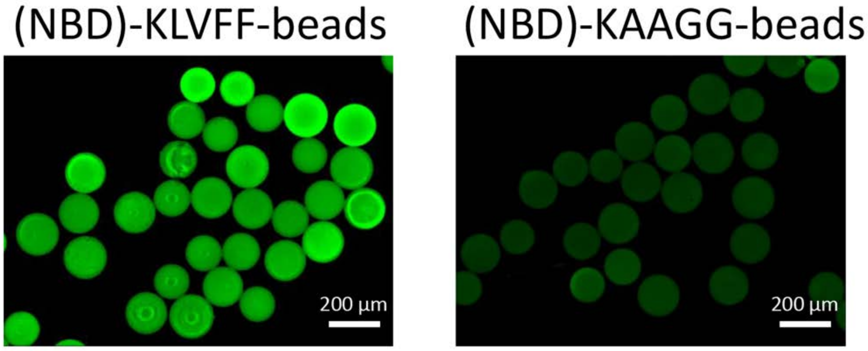

Supplementary Figure 1. Under aqueous condition, TentaGel beads displaying self-assembling peptide KLVFF N-capped by NBD was found to fluoresce strongly. TentaGel beads displaying negative control non-assembling peptide KAAGG did not fluoresce, thus validating the screening assay for self-assembling peptide discovery.

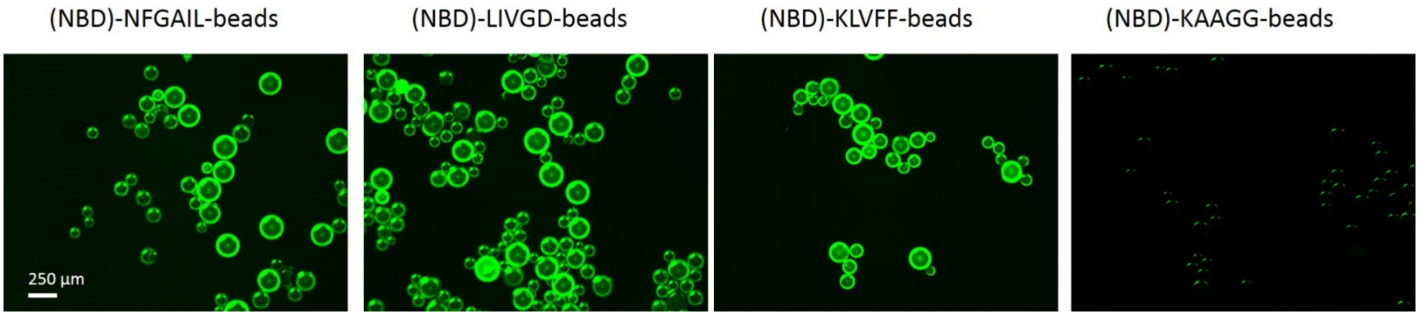

Supplementary Figure 2. Photomicrographs of TentaGel beads displaying NFGAIL, LIVGD, KLVFF, and KAAGG N-terminally capped with NBD at 10% level. NFGAIL, LIVGD, KLVFF are known self-assembling peptides under aqueous condition.

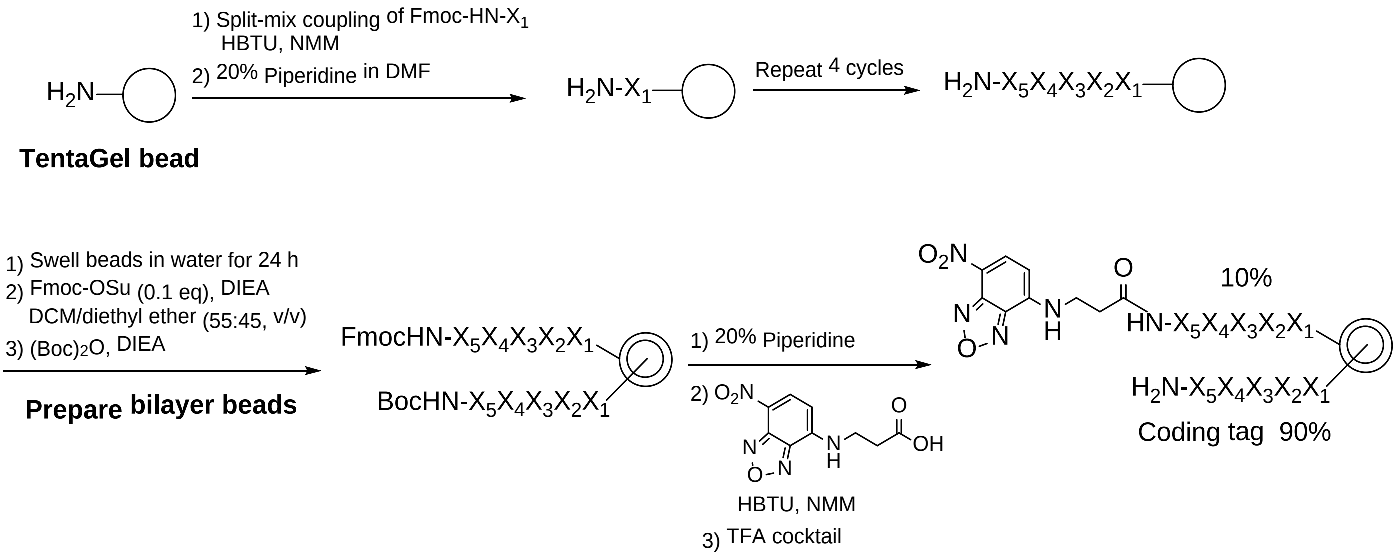

Supplementary Figure 3.. Synthetic scheme of OBOC peptide library N-capped with nitro-1,2,3-benzoxadiazole (NBD), for self-assembling peptide discovery. Only the outer layer of the random peptides was N-capped with the dye. The peptides in the bead interior remained N-terminally free, which is needed for Edman sequencing.

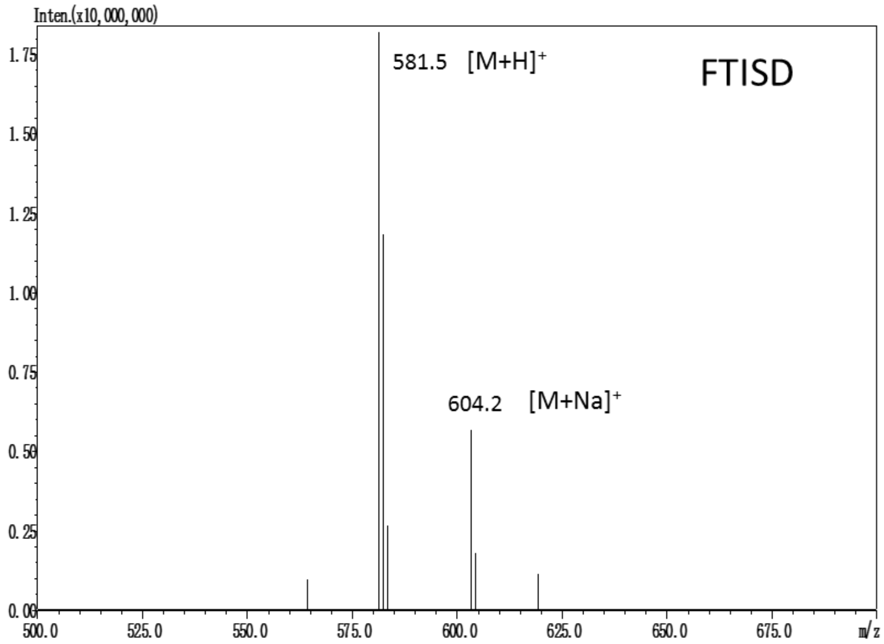

Supplementary Figure 4. The MALDI-TOF spectrum of peptide FTISD

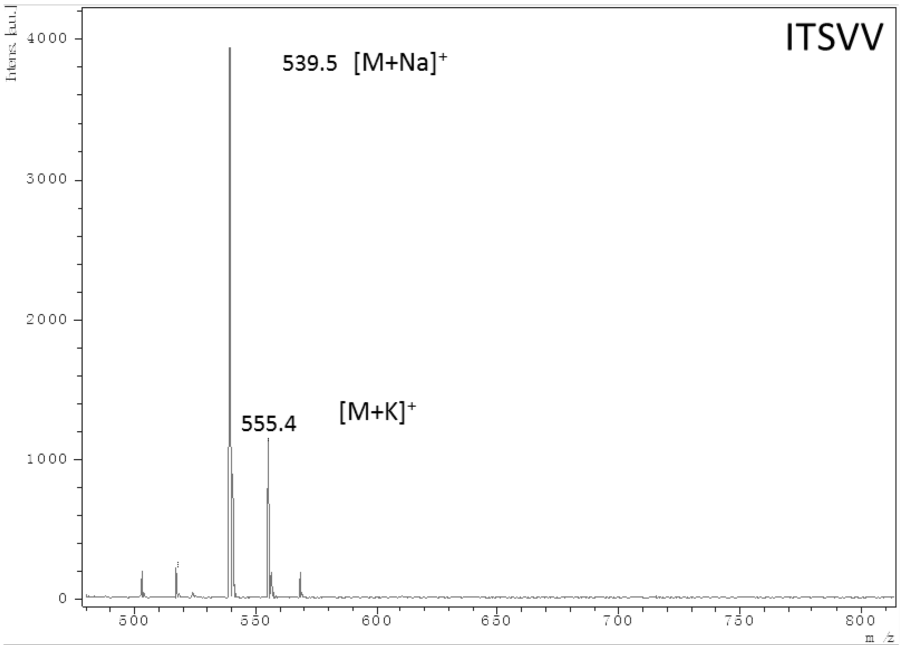

Supplementary Figure 5. The MALDI-TOF spectrum of peptide ITSVV

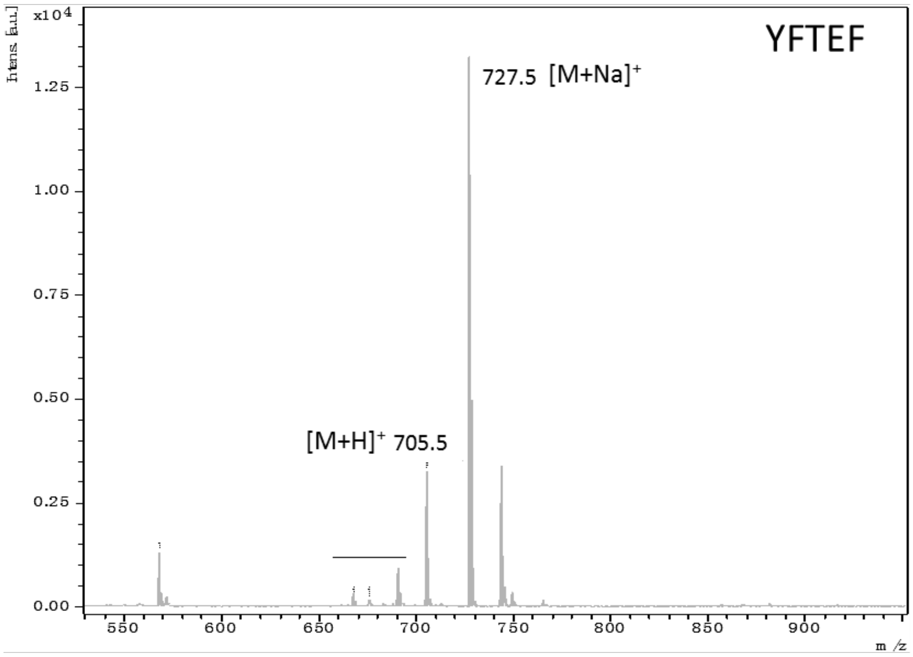

Supplementary Figure 6. The MALDI-TOF spectrum of peptide YFTEF.

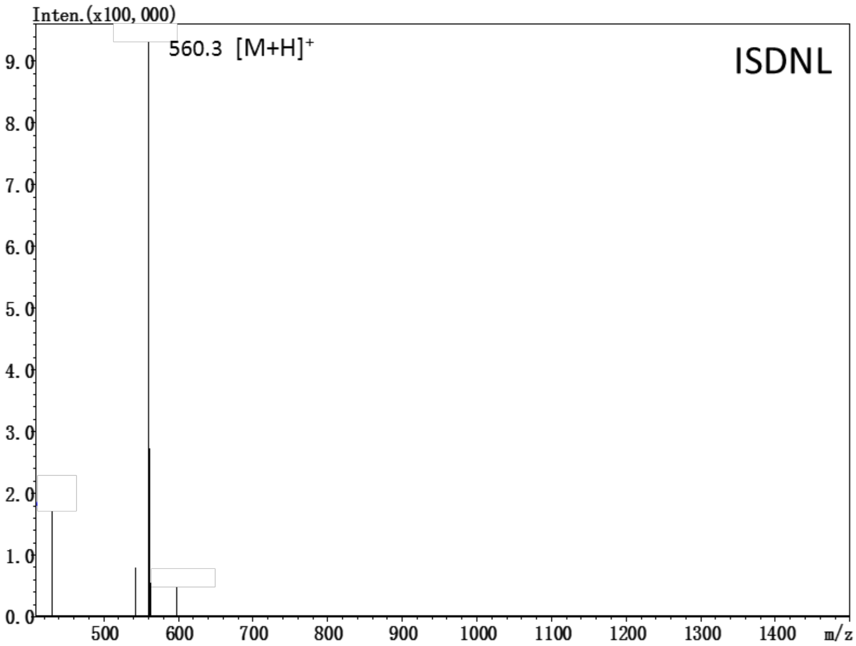

Supplementary Figure 7. The MALDI-TOF spectrum of peptide ISDNL.

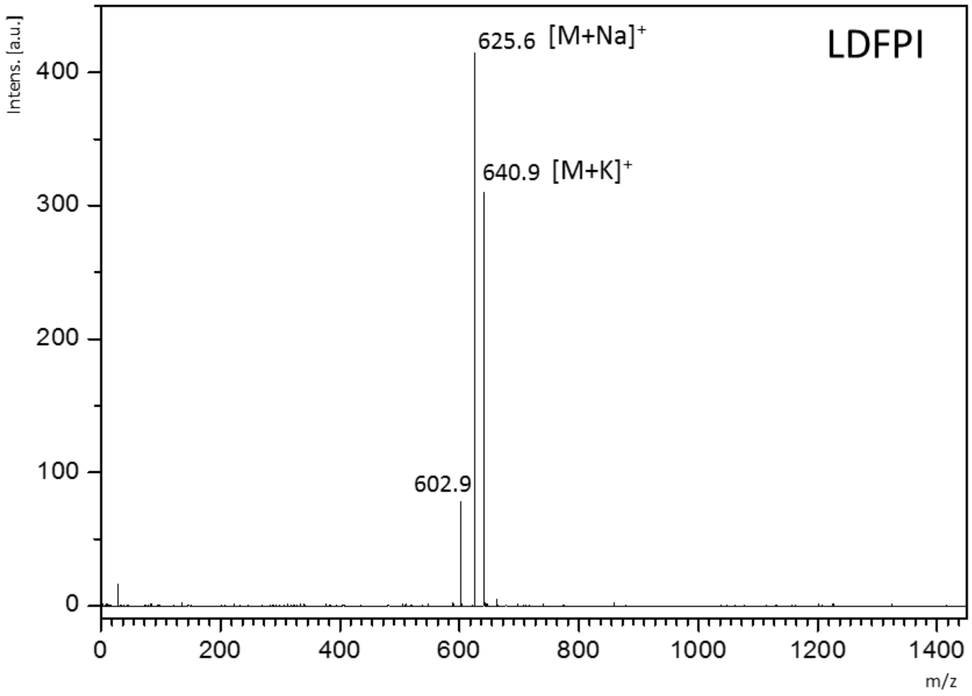

Supplementary Figure 8. The MALDI-TOF spectrum of peptide LDFPI.

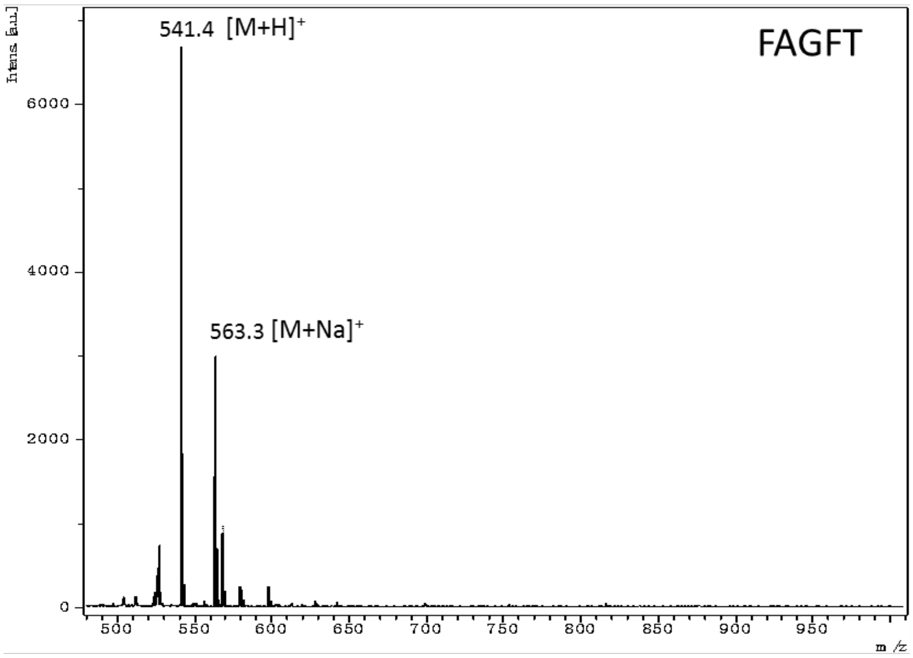

Supplementary Figure 9. The MALDI-TOF spectrum of peptide FAGFT.

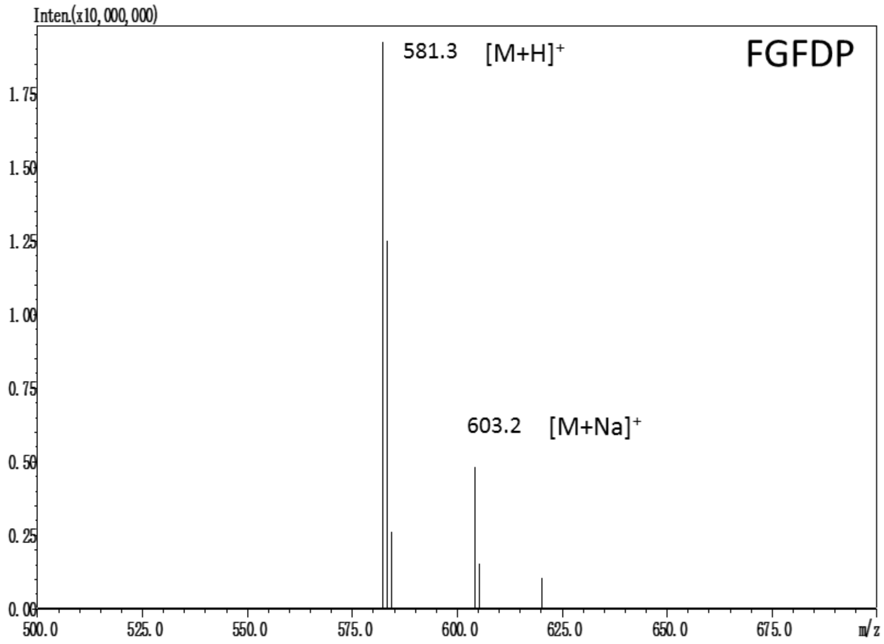

Supplementary Figure 10. The MALDI-TOF spectrum of peptide FGFDP.

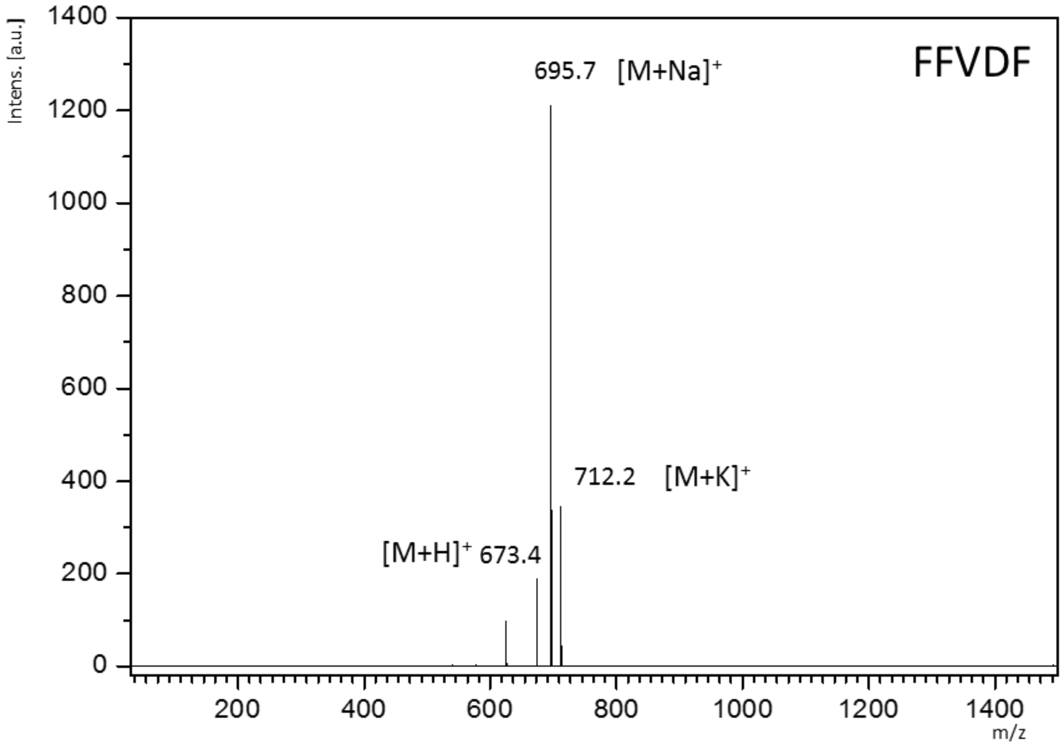

Supplementary Figure 11. The MALDI-TOF spectrum of peptide FFVDF.

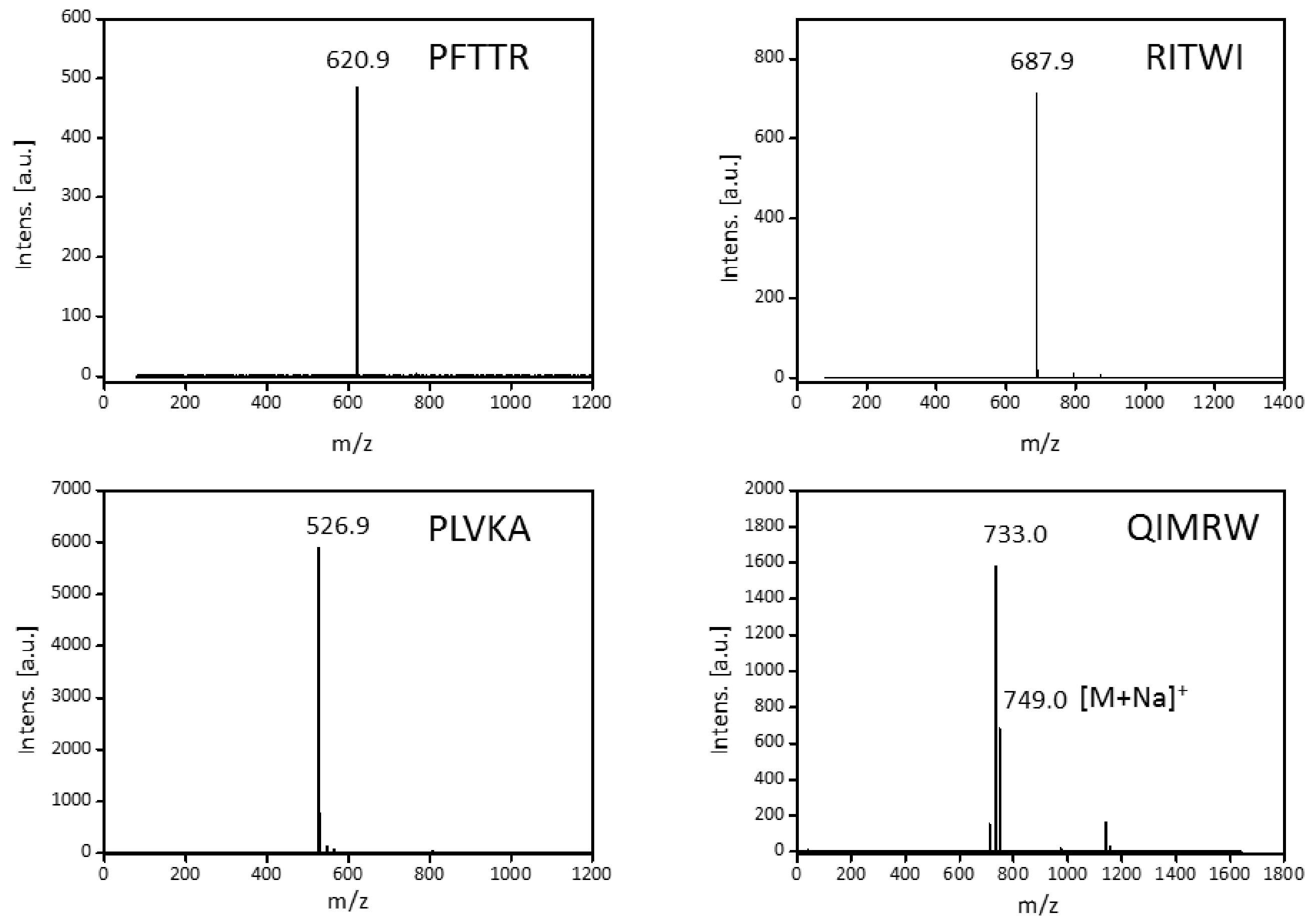

Supplementary Figure 12. The MALDI-TOF spectra of negative non-assembling pentapeptides identified from screening OBOC library.

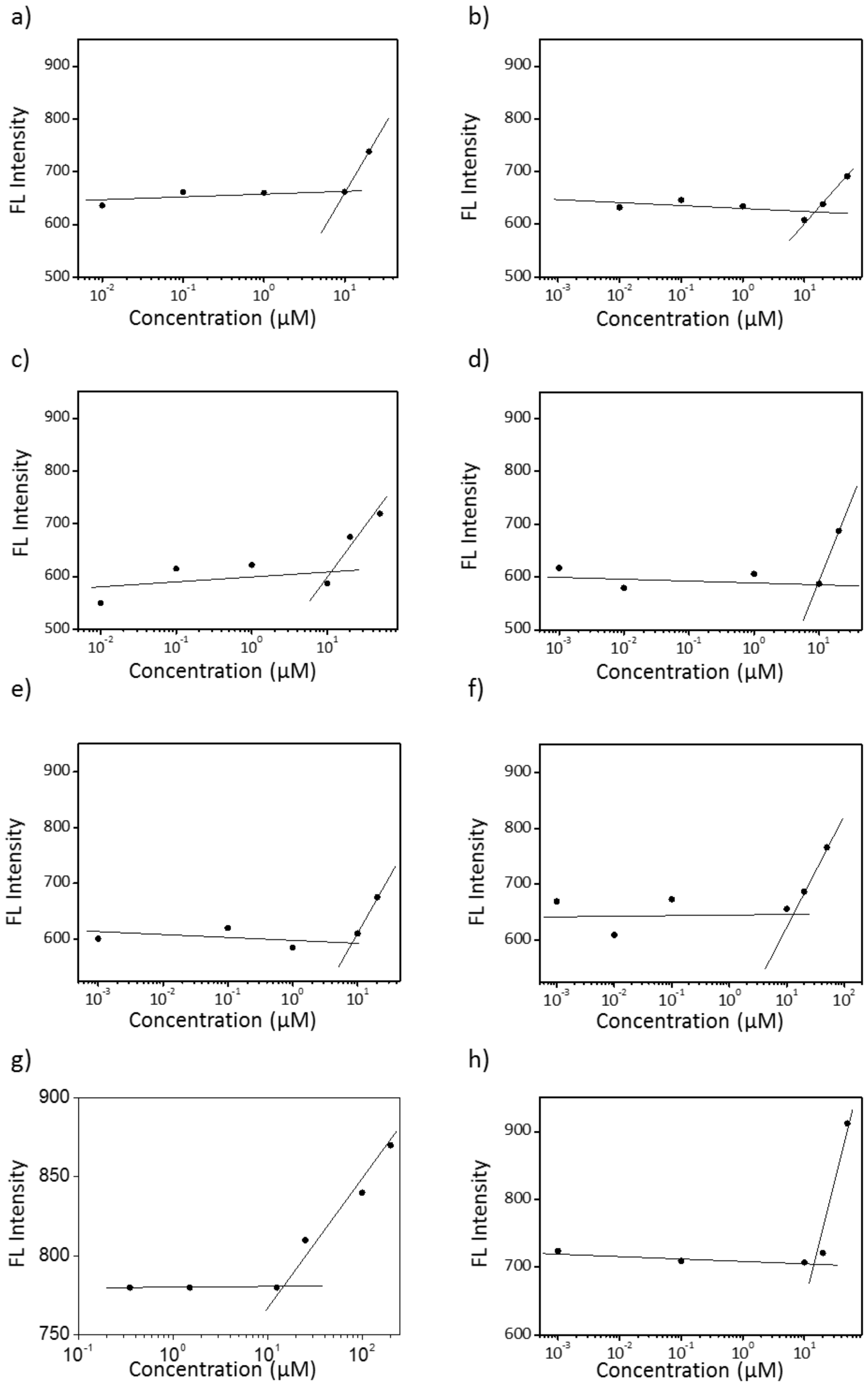

Supplementary Figure 13. The CMC values of the peptide a)-h) FTISD, ITSVV, YFTEF, ISDNL, LDFPI, FAGFT, FGFDP and FFVDF in PBS with 1% DMSO.

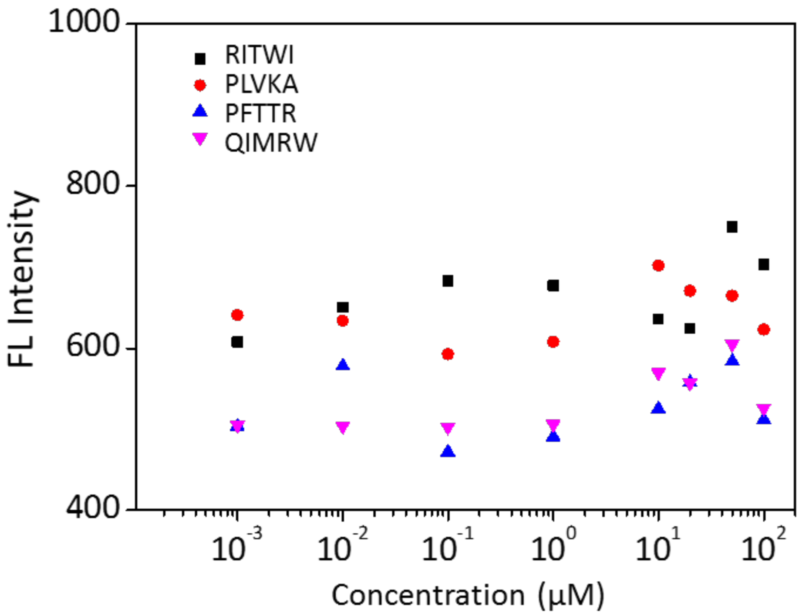

Supplementary Figure 14. The CMC experiment of non-assembling peptide RITWI, PLVKA, PFTTR, and QIMRW in PBS with 1% DMSO.

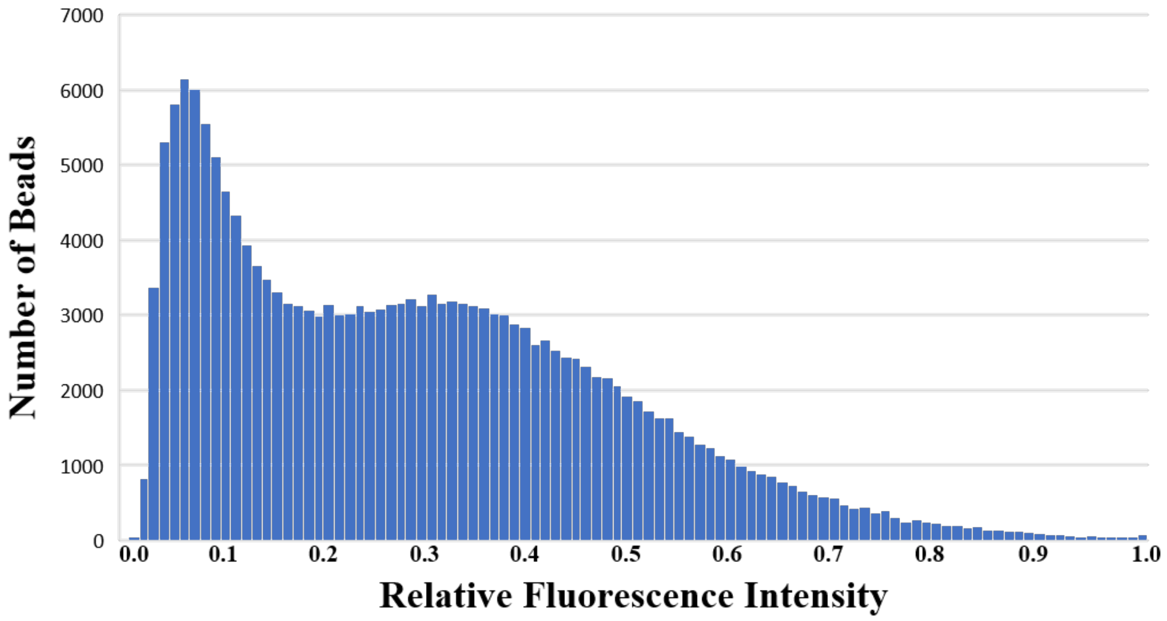

Supplementary Figure 15. Fluorescence profile of an entire immobilized random OBOC pentapeptide library N-capped with NBD dye under aqueous condition, with relative fluorescence unit ranging between 0 to 1.0. Of the 186,288 beads immobilized and screened, 79 or ~0.04% beads displayed a relative fluorescence intensity ≥ 0.99, and 559 or 0.3% beads displayed a relative fluorescence intensity ≥ 0.9. Each vertical bar depicts the number of beads present within a 0.01 relative fluorescence unit range.

Supplementary Table 1. q values, Miller indexes, observed d-spacings (d_{obs}, nm) and calculated d-spacings (d_{cal}, nm) of FFVDF crystals in the nanofibers.

q(nm^{-1})	h	k	l	d_{obs} (nm)	d_{cal} (nm)
1.81	0	1	0	3.47	3.53
2.77	0	0	2	2.27	2.28
3.41	0	1	2	1.84	1.92
5.64	0	0	4	1.11	1.14
7.24	0	4	0	0.87	0.88
8.94	0	4	4	0.70	0.70
10.21	1	3	4	0.61	0.62
11.49	0	2	8	0.55	0.54
13.20	2	0	0	0.48	0.47
13.73	2	0	2	0.46	0.47
14.15	2	2	2	0.44	0.45
15.54	0	8	4	0.40	0.41
15.75	2	4	4	0.40	0.39
17.24	2	4	6	0.36	0.37
20.22	2	8	4	0.31	0.31

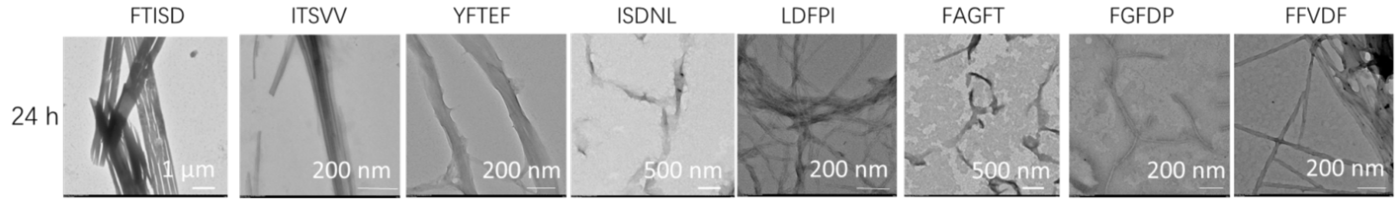

Supplementary Figure 16. The TEM images of pentapeptides FTISD, ITSVV, YFTEF, ISDNL, LDFPI, FAGFT, FGFDP, and FFVDF at 50 µM, 24 h after solubilized in water with 1% DMSO. The scale bar: 1 µm.

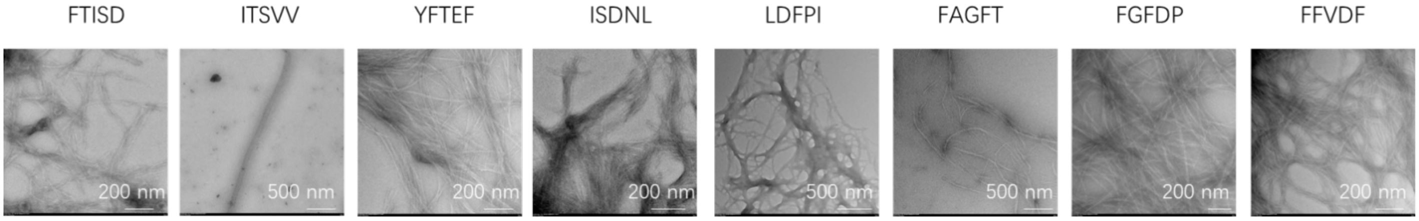

Supplementary Figure 17. The TEM images of pentapeptides FTISD, ITSVV, YFTEF, ISDNL, LDFPI, FAGFT, FGFDP and FFVDF that had undergone thermal annealing at 90 °C for 5 h.

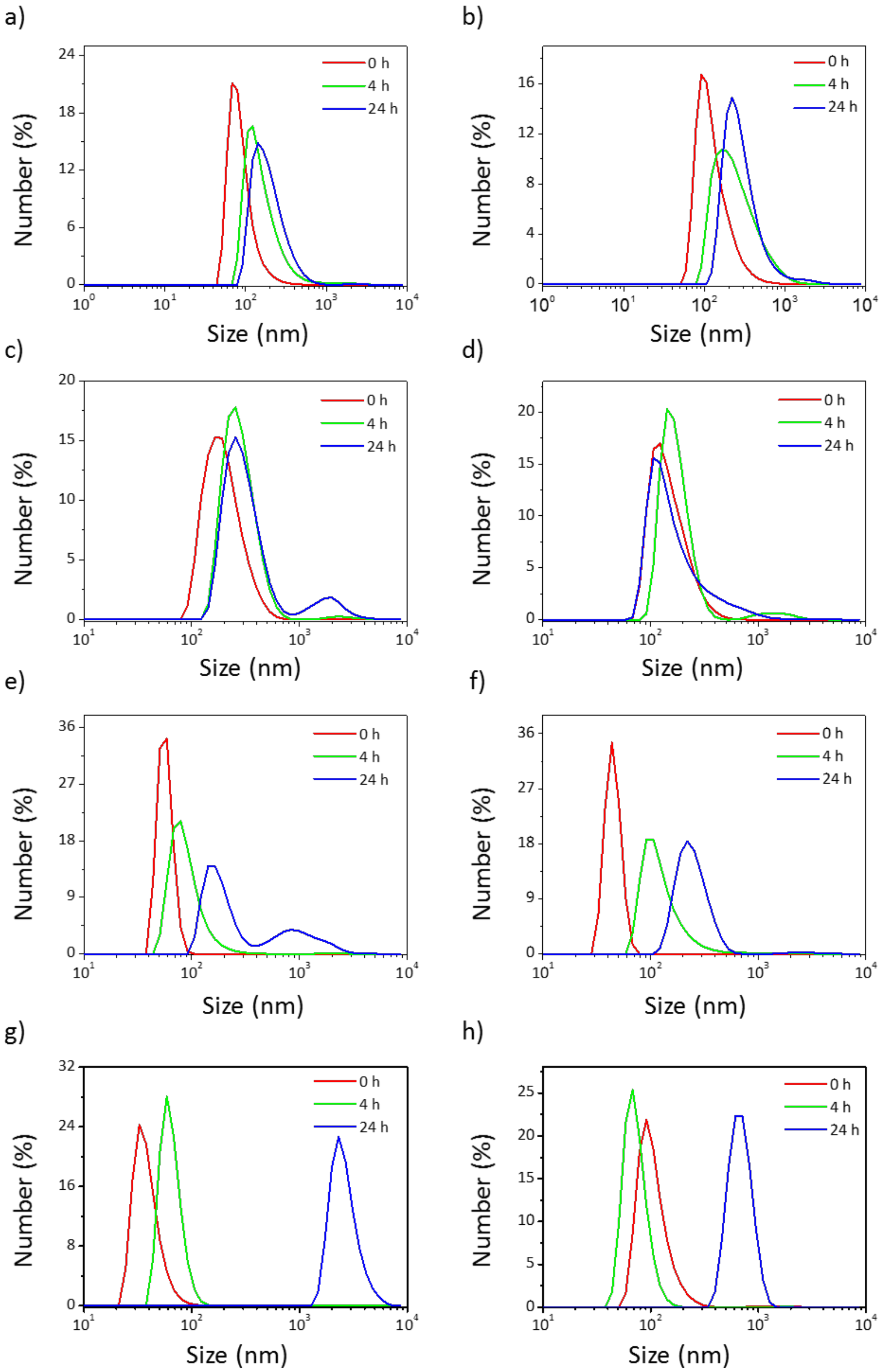

Supplementary Figure 18. The DLS spectra of the pentapeptides a) - h) FTISD, ITSVV, YFTEF, ISDNL, LDFPI, FAGFT, FGFDP, and FFVDF at 50 μM in water with 1% DMSO, obtained at 0, 4 and 24 h.

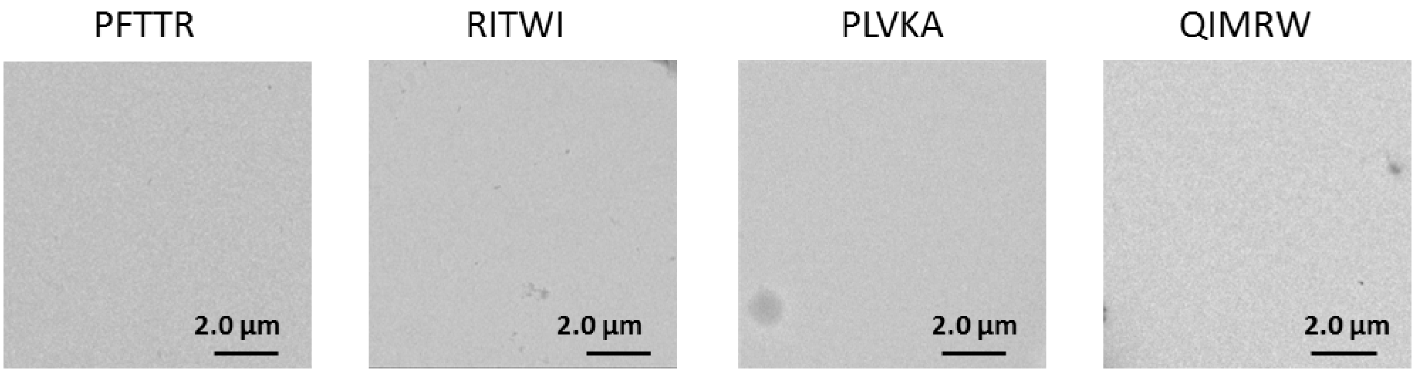

Supplementary Figure 19. The TEM images of the non-assembling pentapeptides PFTTR, RITWI, PLVKA, and QIMRW at 50 µM in water with 1% DMSO after 24 h. No nanostructure was detected.

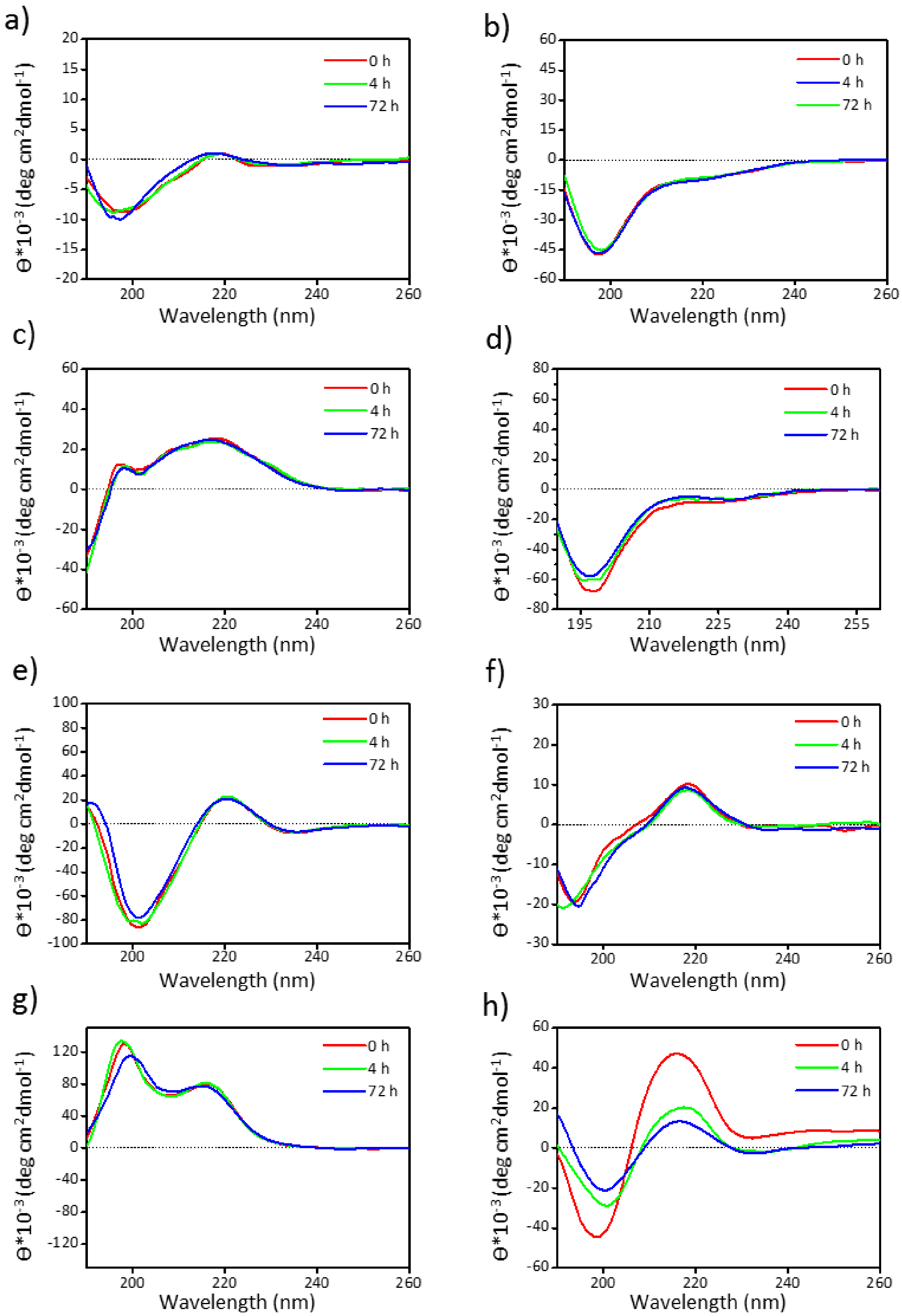

Supplementary Figure 20. The time-dependent CD spectra of the pentapeptides FTISD, ITSVV, YFTEF, ISDNL, LDFPI, FAGFT, FGFDP, FFVDF at 40 μM corresponding to a)-h) in water with 1% DMSO. Significant changes in CD-spectra were observed over time in FFVDF peptide.

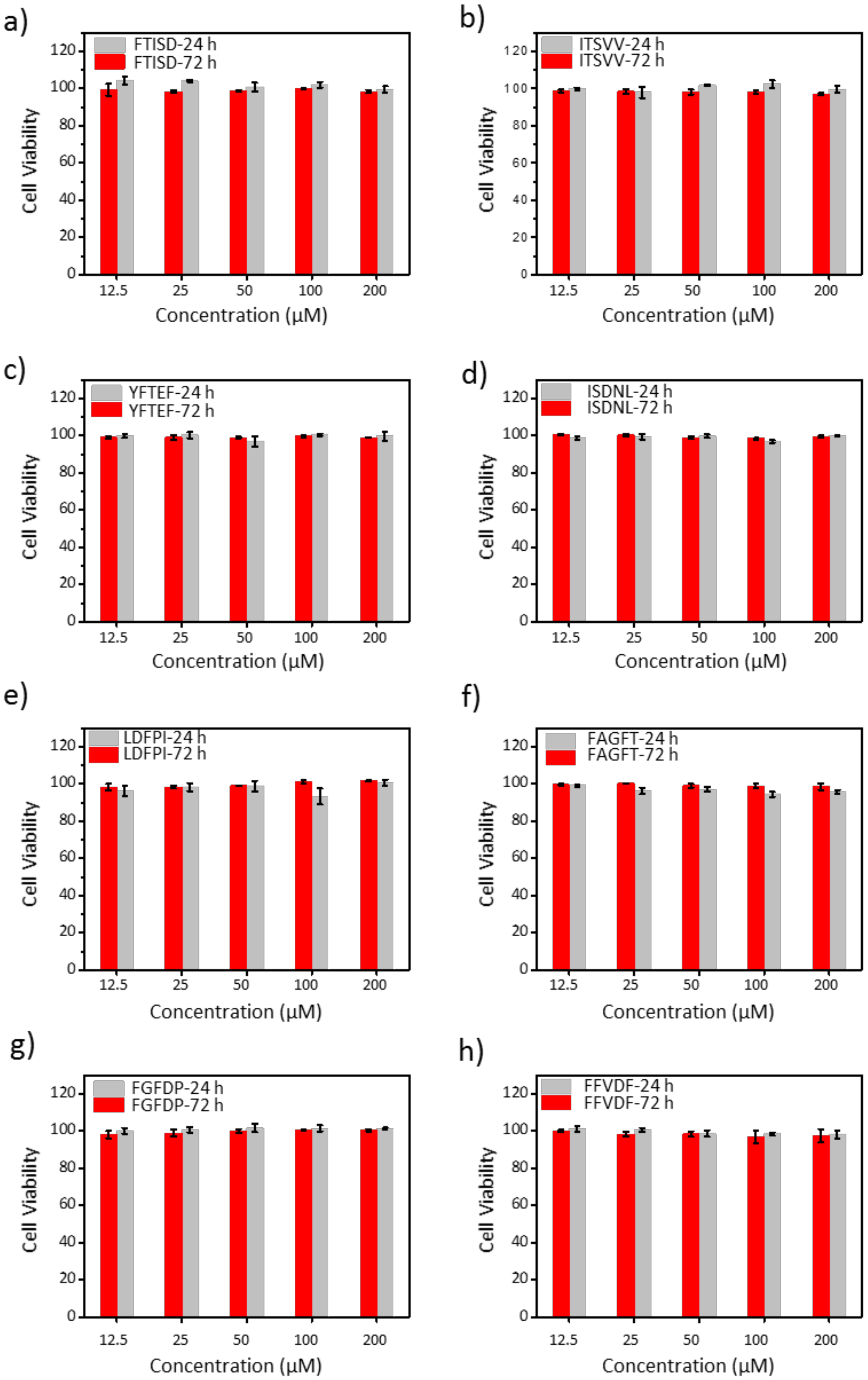

Supplementary Figure 21. a-h. CCK-8 cell viability assay for 8 pentapeptides (FTISD, ITSVV, YFTEF, ISDNL, LDFPI, FAGFT, FGFDP, FFVDF) after incubation with HeLa cells for 24 and 72 h. No cell toxicity was observed, Error bars represent Standard Deviation (n=3).

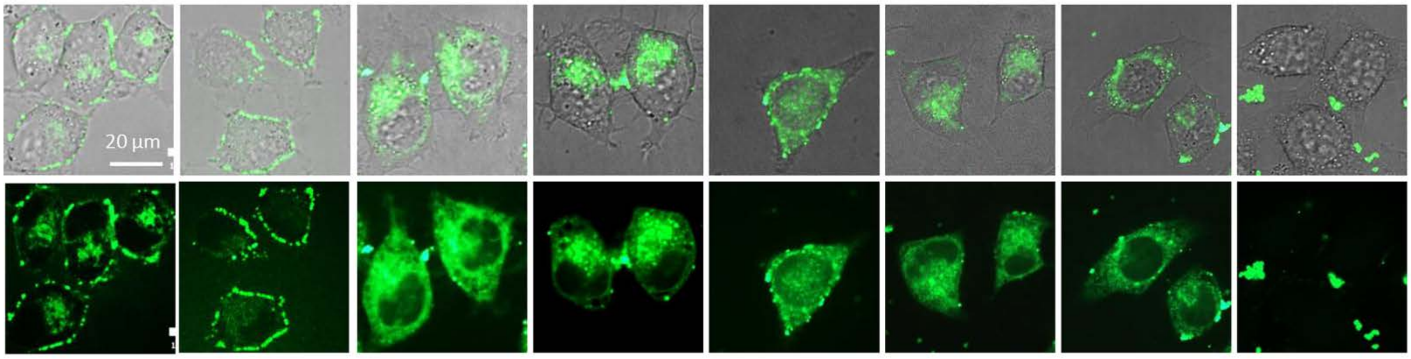

Supplementary Figure 22. Confocal microscopy of HeLa cells after incubation with FITC-labeled pentapeptide assemblies (from left to right: FTISD, ITSVV, YFTEF, ISDNL, LDFPI, FAGFT, FGFDP, FFVDF) for 4 h; peptide concentration used was 50 µM. 3% of the assembled peptides was N-terminally labeled with FITC.

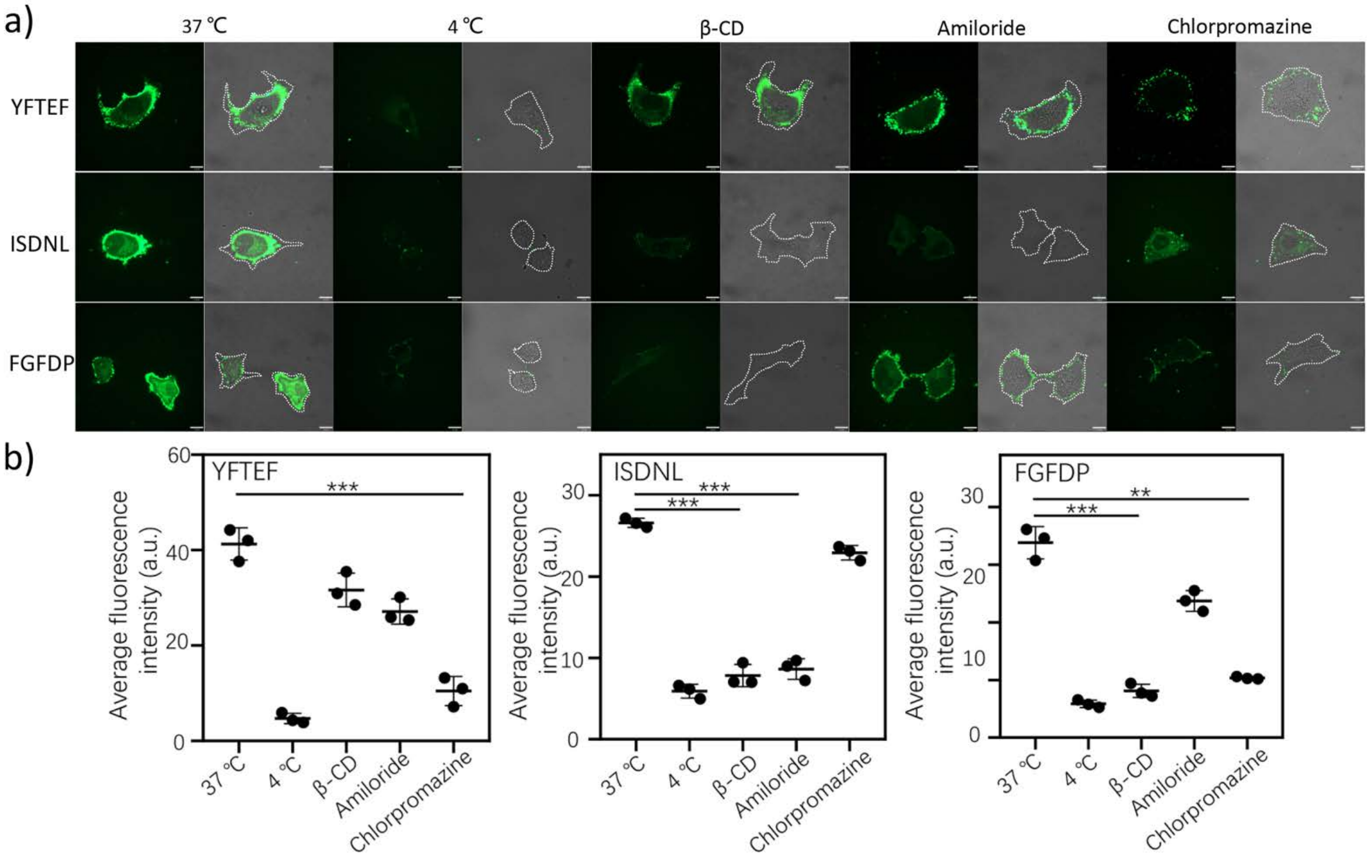

Supplementary Figure 23. Cell uptake pathway analysis for FITC-labeled pentapeptide assemblies YFTEF, ISDNL and FDFDP. a) CLSM images of HeLa cells incubated with pentapeptides (50 µM) at different temperatures (37 or 4 °C) and in the presence of various endocytosis inhibitors, such as amiloride (2 mM), M-β-CD (5 mM), and chlorpromazine (50 µM). The scale bar is 10 µm. b) Relevant quantitative analysis of images from ‘a’. 3% of the assembled peptides was N-terminally labeled with FITC, Error bars represent Standard Deviation (n=3).

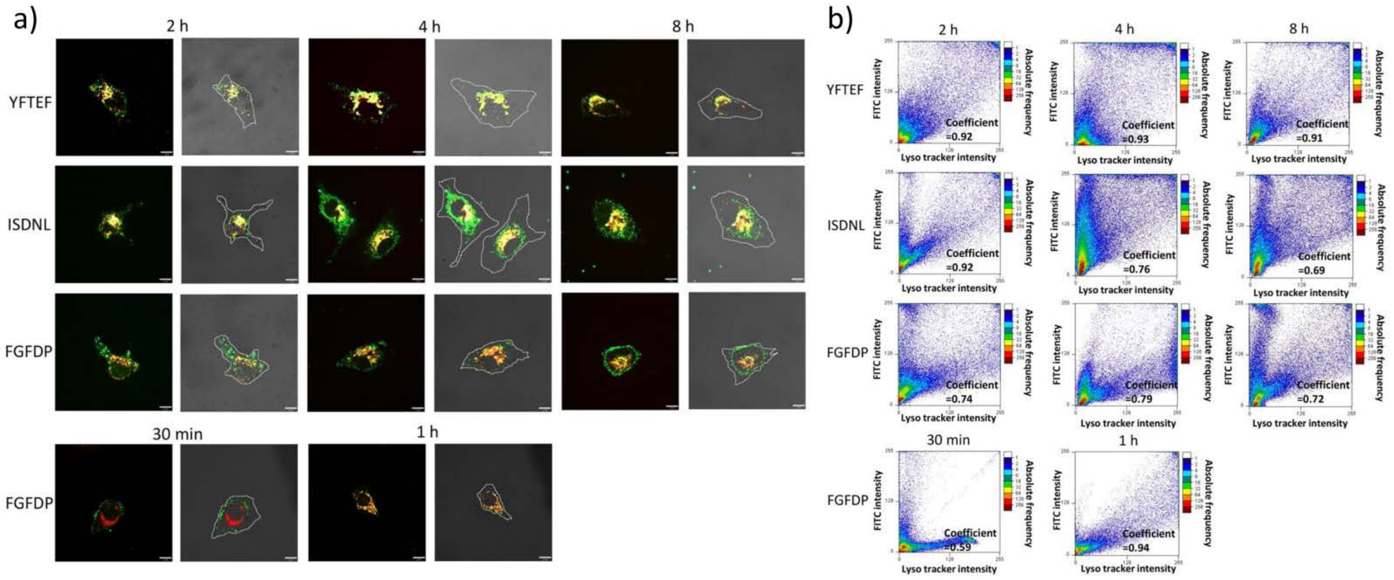

Supplementary Figure 24. a) Time-dependent (2, 4, 8 h) CLSM images for monitoring and quantitatively analyzing uptake of fluorescent peptide assemblies (YFTEF, ISDNL, and FGFDP at 50 µM) by HeLa cells. The scale bar is 10 µm. b) Corresponding colocalization analysis of images from 'a'.

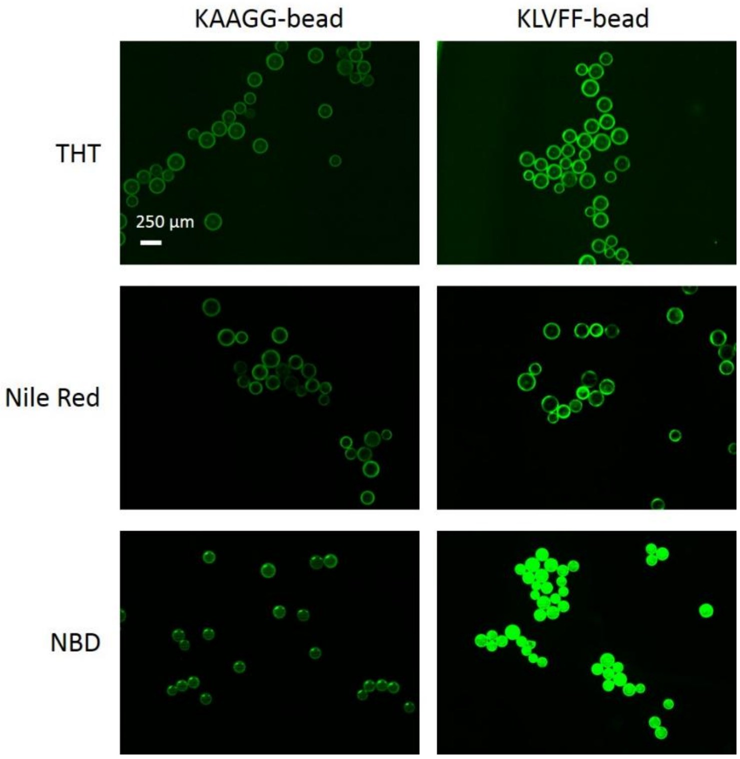

Supplementary Figure 25. Fluorescent microscopy of KLVFF and KAAGG beads after incubation with free ThT, Nile Red, or NBD in water for 2 hours.
